# Detection of the mecA Gene and Its Association With Antimicrobial Resistance Among Coagulase-Negative Staphylococci Isolated From Clinical Samples in a Tertiary Care Hospital: A Cross-Sectional Study

**DOI:** 10.7759/cureus.81643

**Published:** 2025-04-03

**Authors:** Vanlal Tluanpuii, Rakesh K Mahajan

**Affiliations:** 1 Microbiology, Atal Bihari Vajpayee Institute of Medical Sciences and Dr. Ram Manohar Lohia Hospital, New Delhi, IND

**Keywords:** antimicrobial resistance‎, antimicrobial resistance pattern, coagulase-negative staphylococci, community acquired infection, meca gene, methicillin resistance, methicillin resistant coagulase-negative staphylococcus, public health, sccmec, staphylococcal infections

## Abstract

Introduction

Coagulase-Negative Staphylococci (CoNS) have become major pathogens causing a variety of infections. Antimicrobial resistance, brought about by the acquisition of the *mec*A gene, further exacerbates this burden. This gene encodes the altered membrane-bound protein called penicillin-binding protein (PBP2a), which lowers the binding affinity for β-lactam antibiotics. The widespread distribution of this gene has led to the surge of methicillin-resistant CoNS (MR-CoNS). Therefore, this study aimed to detect the prevalence of the *mec*A gene and its correlation with antimicrobial resistance.

Methods

This was a cross-sectional observational study conducted over a period from January 2021 to May 2022 at a tertiary care hospital in Delhi, India. A total of 100 pure isolates of CoNS from various samples were included and identified at the species level using conventional methods and an automated Vitek-2 ID system (bioMérieux, Marcy-l'Étoile, France). Antibiotic susceptibility testing was performed using the Kirby-Bauer disk diffusion method, as per the Clinical and Laboratory Standards Institute (CLSI) 2020 guideline, with the following antibiotics: cefoxitin (30 μg), erythromycin (15 μg), cotrimoxazole (1.25/23.75 μg), tetracycline (30 μg), gentamicin (10 μg), and ciprofloxacin (5 μg). The minimum inhibitory concentration (MIC) for vancomycin was detected using the Epsilometer test (E-test). All of the 100 isolates were also subjected to real-time polymerase chain reaction (rt-PCR) for the detection of the *mec*A gene.

Results

Out of 100 CoNS isolates, *Staphylococcus haemolyticus* was the most commonly isolated, accounting for 44%. Cefoxitin was sensitive for only 20% of the isolates. Sensitivities for ciprofloxacin, cotrimoxazole, gentamicin, tetracycline, and erythromycin were 56%, 55%, 50%, 49%, and 25%, respectively. All isolates were sensitive to vancomycin. Out of 100 CoNS isolates that were subjected to *mec*A gene detection by rt-PCR, 82 (82%) isolates were positive for the *mec*A gene, and the rest were negative. Among the 82 (82%) *mec*A gene-positive isolates, seven (8.5%) were found to be cefoxitin sensitive, and among the 18 (18%) that were negative for the *mec*A gene, cefoxitin resistance was detected in five (27.7%) isolates by disk diffusion method. Both cefoxitin disk diffusion and *mec*A gene detection by rt-PCR were employed to detect methicillin resistance among the CoNS isolates.

Conclusion

The *mec*A gene was detected in 82 (82%) isolates. However, some *mec*A-positive CoNS were found to be susceptible to cefoxitin by the disk diffusion method, while some *mec*A-negative CoNS isolates were resistant. This suggests that no single diagnostic approach is capable of identifying methicillin resistance alone. Therefore, *mec*A gene detection must be implemented along with cefoxitin disk diffusion to make accurate and early therapeutic decisions.

## Introduction

Coagulase-Negative Staphylococci (CoNS) are gram-positive cocci that have ubiquitous distribution and frequently colonize the skin and mucous membranes of humans. CoNS were historically considered isolates with little or questionable pathogenic potential, but the significance of CoNS became apparent in the late 1970s when the involvement of CoNS species was reported in various invasive diseases, such as osteomyelitis, otitis media, endophthalmitis, urinary tract infections, meningitis, pneumonia, and indwelling device-related and prosthetic implant infections [1,2]. The systemic infections are usually the result of colonizing *Staphylococcus* species gaining entry into the bloodstream. The main pathogenic role of CoNS is attributed to their ability to anchor to biotic and abiotic surfaces, forming a biofilm [3]. CoNS are typical opportunistic organisms that not only colonize immunocompromised patients but also healthy populations and are considered one of the major hospital pathogens, with an ever-increasing impact on human life and health.

Penicillin has been the consensus first-line antibiotic to treat Staphylococcal infections, but it was soon replaced as the first-line antibiotic due to the development of a powerful resistance mechanism in the form of β-lactamase production [1,2]. The major resistance concern in *Staphylococcus* species emerged when it acquired a single genetic element called the Staphylococcal cassette chromosome *mec* (SCC*mec*), containing the *mec*A gene [4,5]. This gave rise to the emergence of notorious methicillin-resistant strains, which rendered β-lactams, monobactams, and carbapenems completely ineffective, leaving the limited option of vancomycin to treat Staphylococcal infections [6,7]. This gene encodes the altered membrane-bound protein, the so-called penicillin-binding protein 2a (PBP2a), that decreases the binding affinity for β-lactam antibiotics [6,7]. The widespread distribution of this gene has led to the surge of methicillin-resistant strains of *Staphylococcus* species, such as methicillin-resistant CoNS (MR-CoNS) as well as methicillin-resistant *Staphylococcus aureus* (MRSA) [4].

Since methicillin resistance acquired by CoNS threatens the effective prevention and intervention of therapeutic strategies aimed at both community- and hospital-acquired infections, this work was undertaken to study the prevalence of the *mec*A gene among CoNS and its correlation with antimicrobial resistance.

## Materials and methods

This was a cross-sectional observational study conducted from January 2021 to May 2022 at a tertiary care hospital in Delhi, India. A total of 100 pure isolates of CoNS from various clinical samples, such as blood, pus, urine, body fluids (cerebrospinal fluid, pleural fluid, synovial fluid, etc.), and indwelling devices were included in the study and processed as per Standard Microbiological Techniques in the Microbiology Department. CoNS that were growing in mixtures were excluded from the study.

All CoNS isolates were identified at the species level by conventional methods, such as colony morphology, gram stain, catalase test, slide and tube coagulase test, sugar fermentation reactions, etc., as well as the automated Vitek-2 ID system (bioMérieux, Marcy-l'Étoile, France). All isolates were tested against cefoxitin (30 μg), erythromycin (15 μg), cotrimoxazole (1.25/23.75 μg), tetracycline (30 μg), gentamicin (10 μg), and ciprofloxacin (5 μg) by Kirby-Bauer disk diffusion testing, as per the Clinical and Laboratory Standards Institute (CLSI) 2020 guidelines. The minimum inhibitory concentration (MIC) for vancomycin was detected using the Epsilometer test (E-test). Cefoxitin (30 μg) disk was used as a surrogate marker for the detection of *mec*A-mediated methicillin resistance in CoNS. A 0.5 McFarland standard suspension of the isolate was made, and the lawn was cultured on a Mueller-Hinton agar (MHA) plate. Plates were incubated at 37°C for 24 hours, and zone diameters were measured. An inhibition zone diameter of ≤24 mm for cefoxitin was reported as resistant, and ≥25 mm as sensitive.

Real-time polymerase chain reaction (rt-PCR) was performed on all isolates for the detection of the *mec*A gene. Extraction of nucleic acids from the freshly subcultured and isolated bacterial colonies was performed using the NUCLISENS® EASYMAG® Automated Extraction System (bioMérieux, Marcy-l'Étoile, France). The Microbial DNA qPCR Assay Kit by Qiagen, Hilden, Germany, was used for the detection of the *mec*A gene. PCR reaction mixtures were prepared as per the instructions given by Qiagen, under laminar flow, with strict precautions to prevent cross-contamination. Amplification of the gene was performed with a 10-minute initial PCR activation step at 95°C, followed by one cycle of denaturation for 15 seconds at 95°C and annealing and extension for two minutes each at 60°C for a total of 40 cycles.

The study was approved by the Institutional Ethics Committee of Atal Bihari Vajpayee Institute of Medical Sciences and Dr. Ram Manohar Lohia Hospital, New Delhi, India (approval No. TP(MD/MS)(72/2020)/IEC/ABVIMS/RMLH/312).

## Results

Out of 100 CoNS isolates, 50 (50%) were isolated from blood, 20 (20%) from urine, 14 (14%) from pus samples, 10 (10%) from aspirated body fluids, and the remaining six (6%) from indwelling devices.

All the 100 isolates were distributed over a total of five species, viz., *Staphylococcus haemolyticus* (44%), *Staphylococcus epidermidis* (32%), *Staphylococcus saprophyticus* (10%), *Staphylococcus hominis* (9%), and the remaining five isolates (5%) as *Staphylococcus warneri*. A total of 44 (44%) of CoNS isolations were from patients admitted to Intensive Care Units (ICUs), followed by 39 (39%) admitted to wards, and 17 (17%) from patients attending Outpatient Departments (OPDs). 

**Figure 1:**
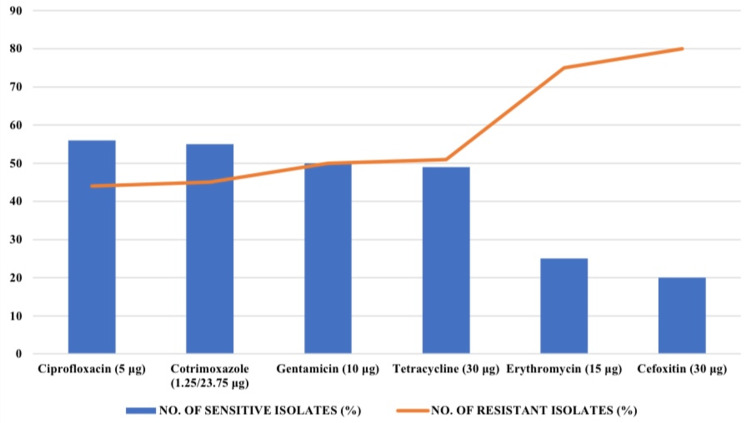
Antibiotic susceptibility pattern of CoNS isolates. ConS, Coagulase-negative staphylococci

In the cefoxitin (30 µg) disk diffusion test, only 20 out of a total of 100 isolates were sensitive to cefoxitin. Sensitivities for ciprofloxacin, cotrimoxazole, gentamicin, tetracycline, and erythromycin were 56% (56), 55% (55), 50% (50), 49% (49), and 25% (25%), respectively, as shown in Figure 1. Meanwhile, all isolates were sensitive to vancomycin, with an MIC of less than 4 µg/mL. 

**Figure 2:**
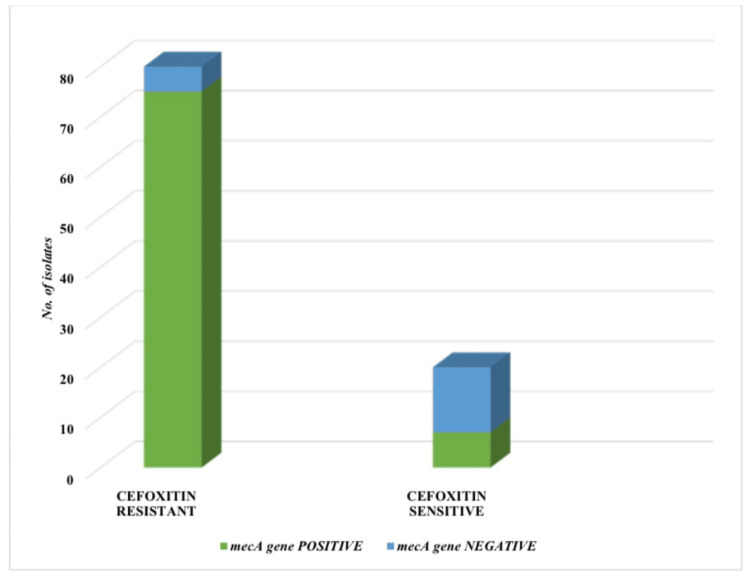
Graph showing the correlation of methicillin resistance detection by cefoxitin (30 μg) disk and the presence of the mecA gene.

Out of 100 CoNS isolates that were subjected to *mec*A gene detection by rt-PCR, 82 (82%) were positive for the *mec*A gene, and 18 (18%) were negative for the *mec*A gene. Among the 82 (82%) *mec*A gene-positive isolates, seven (8.5%) were found to be cefoxitin sensitive, and among the 18 (18%) that were negative for the *mec*A gene, cefoxitin resistance was detected in five (27.7%) isolates in the disk diffusion test. Both cefoxitin disk diffusion and the *mec*A gene detection by rt-PCR were used to detect methicillin resistance in CoNS. Table 1 and Figure 2 show the correlation between methicillin resistance detection by cefoxitin disk diffusion and the presence of the *mec*A gene. 

**Table 1:** Correlation of methicillin resistance detection by cefoxitin (30 μg) disk and presence of mecA gene.

Disk diffusion method	*mec*A gene positive (No. of isolates)	*mec*A gene negative (No. of isolates)	Total
Cefoxitin resistant (No. of isolates)	75	5	80
Cefoxitin sensitive (No. of isolates)	7	13	20
Total	82	18	100

## Discussion

CoNS have remarkably evolved through the years and emerged as important nosocomial pathogens [5]. Currently, more than 70% of CoNS circulating worldwide are methicillin-resistant [2]. The presence of the *mec*A gene results in treatment failures with β-lactam antibiotics, one of the most important classes of antimicrobials against gram-positive organisms. Therefore, early and accurate detection of patterns of resistance is an absolute necessity to introduce appropriate therapeutic interventions.

In this study, the isolation rate of CoNS species was highest from blood samples (50%) and least from indwelling devices (6%). Two studies by Bhatt et al. and Mane et al. from India have reported similar findings, suggesting a possible significant role of CoNS in systemic infections [8,9]. In contrast, the work of Williams et al. has logged the maximum isolations from indwelling devices, accounting for 62.5% [10]. The differences in the results of various studies could have been due to differences in the quality and type of medical services provided, and non-uniformity in study designs. Nevertheless, the significance of CoNS as an established cause of bacteremia appears increasingly significant [1,7]. The surge in the incidence of CoNS bacteremia may be due to an ever-expanding pool of susceptible populations, such as chronically ill, morbid, elderly patients, and immunocompromised patients, as well as the increased use of antibiotics and foreign implantable devices, such as indwelling and insertion devices.

In this study, 44% of CoNS isolations were from intensive care areas, followed by 39% and 17% from patients in wards and OPDs, respectively. Similar findings of 45.5% and 48.7% isolations of CoNS from ICUs have been reported [5,8]. The large prevalence of CoNS in critical care settings occurs due to their ability to attach to abiotic surfaces, like central venous catheter tips and endotracheal tubes used in critically ill patients [11,12].

Five different species were identified in this study based on results from conventional identification techniques and the Vitek-2 automated ID system. Of the 100 CoNS isolates, 44 (44%) were identified as *S. haemolyticus*, followed by 32 (32%) *S. epidermidis*, 10 (10%) *S. saprophyticus*, 9 (9%) *S. hominis*, and 5 (5%) *S. warneri*. Our findings of the predominance of *S. haemolyticus* corroborate with those of Jain et al. and Al-Tamimi et al., who have described 58% and 47% isolations of *S. haemolyticus*, respectively [12,13]. Multiple *Staphylococcus* species have been reported all over the world [14]. Conversely, several studies have reported *S. epidermidis* as the most common species [15,16]. The findings of these studies may suggest differences in the geographical distribution of different species of CoNS [17,18].

In the present study, 80 (80%) of the CoNS isolates were resistant to cefoxitin. Similar to our study, Singh et al. and Manoharan et al. have reported cefoxitin resistance rates of 72.5% and 91.3%, respectively [17,18]. In contrast, one study found only 25% resistance to cefoxitin [8]. The high frequency of cefoxitin resistance seen in this study could potentially be attributed to the fact that the majority of samples were from patients admitted to ICUs and wards, where these patients may be empirically exposed to multiple classes of antimicrobials [2]. The widespread occurrence of resistance among CoNS may be due to the possibility of genetic exchange of resistance markers between different CoNS species [7,19]. Besides cefoxitin, other antibiotics like ciprofloxacin, cotrimoxazole, gentamicin, tetracycline, and erythromycin showed sensitivities of 56% (56%), 55% (55%), 50% (50%), 49% (49%), and 25% (25%), respectively. All isolates (100%) were sensitive to vancomycin, with an MIC of less than 4 μg/mL. 

The *mec*A gene is one of the most established causes of methicillin resistance in *Staphylococcus* species [3]. *mec*A gene detection by rt-PCR is considered the gold standard. In the present study, a total of 82 (82%) CoNS were found to be positive for the *mec*A gene. The high prevalence of the *mec*A gene in our study appears to agree with other studies done by a few authors [20-22]. A higher prevalence than in this present study was also seen in a study by Hira et al., where the *mec*A gene was observed in 87% of CoNS isolates [23]. In contrast to the present study, the low prevalence of the *mec*A gene has also been reported by other authors [24-26].

However, in this current study, inconsistency in the number of cefoxitin-resistant CoNS and the presence of the *mec*A gene was found. Five CoNS isolates that were negative for the *mec*A gene showed cefoxitin resistance in the disk diffusion method, and seven CoNS isolates that were positive for the *mec*A gene were cefoxitin-sensitive by the disk diffusion method. Similar discordant findings have also been published by other workers [8,14]. It has been reported that resistance to cefoxitin in *mec*A-negative strains could be attributed to the hyperproduction of β-lactamases [2,27]. Manoharan et al. have described the *mec*C gene as another important gene that may cause methicillin resistance [18]. Hososaka et al. have also suggested that resistance could be expressed by other genes, like *bla*I, *bla*RI, *bla*Z, *mec*I, and *mec*RI, other than the *mec*A gene [14]. However, since this study was designed to detect only the *mec*A gene, it was not possible to explore the details of the reason for these aberrant results. Nevertheless, the findings of the above-mentioned studies clearly spell out a variety of mechanisms that may be participating in the development of methicillin resistance. 

Another important finding was that seven *mec*A gene-positive CoNS isolates were sensitive to cefoxitin in the disk diffusion test. Williams et al. have hypothesized the possibility of mutations in genes known as factors essential for methicillin resistance (FEM) or auxiliary factors [10]. It is believed that mutations in these genes, especially *fem*AB, may lead to hyper-susceptibility to β-lactam antibiotics, even with the continuous production of altered PBPs encoded by the *mec*A gene [10].

Therefore, more prospective, structured research is needed to fully comprehend the genetic mechanisms of methicillin resistance, in order to identify the appropriate targets for controlling the spread of this deadly drug resistance in this important hospital pathogen. It may also aid in the development of novel antibiotics that are more targeted at the various potential mechanisms of resistance. 

## Conclusions

This study found that *mec*A-positive CoNS were responsive to cefoxitin by disk diffusion method, although certain *mec*A-negative CoNS isolates were resistant to it. This implies that multiple mechanisms contribute to the expression of methicillin resistance and that no single diagnostic approach can detect it alone. Therefore, it is essential to detect the *mec*A gene in conjunction with the disk diffusion protocol to accurately identify methicillin resistance, ensuring that no isolate of MR-CoNS is overlooked and that the appropriate antibiotic is started on time.
